# New quantitative blood flow assessment of gastric conduit with indocyanine green fluorescence in oesophagectomy: prospective cohort study

**DOI:** 10.1093/bjsopen/zraf135

**Published:** 2025-12-01

**Authors:** Daisuke Kajiyama, Yuto Kubo, Takashi Shigeno, Kazuma Sato, Naoto Fujiwara, Hiroyuki Daiko, Takeo Fujita

**Affiliations:** Division of Esophageal Surgery, National Cancer Center Hospital East, Kashiwa, Japan; Course of Advanced Clinical Research of Cancer, Juntendo University Graduate School of Medicine, Tokyo, Japan; Division of Esophageal Surgery, National Cancer Center Hospital East, Kashiwa, Japan; Department of Gastrointestinal Surgery, Institutive of Science Tokyo, Tokyo, Japan; Division of Esophageal Surgery, National Cancer Center Hospital East, Kashiwa, Japan; Department of Esophageal Surgery, Tokyo Metropolitan Komagome Hospital, Tokyo, Japan; Division of Esophageal Surgery, National Cancer Center Hospital, Tokyo, Japan; Division of Esophageal Surgery, National Cancer Center Hospital East, Kashiwa, Japan; Course of Advanced Clinical Research of Cancer, Juntendo University Graduate School of Medicine, Tokyo, Japan

## Abstract

**Background:**

Anastomotic leakage (AL) remains a critical complication following oesophagectomy, with inadequate perfusion of the conduit identified as a major contributing factor. Although indocyanine green (ICG) fluorescence angiography has been used intraoperatively to assess conduit blood flow, the clinical utility of objective ICG fluorescence indicators for anastomotic site determination has not been well established. This study investigated the association between ICG fluorescence intensity, measured using a new quantitative blood flow assessment technique, and the incidence of AL in patients undergoing gastric conduit reconstruction.

**Methods:**

Prospective analysis of patients who underwent subtotal oesophagectomy with gastric conduit reconstruction between July 2023 and May 2024. Intraoperative real-time perfusion was assessed using the SPY-PHI imaging system in conjunction with SPY-QP software. Quantitative fluorescence intensity measurements were obtained at the terminal branch of the right gastroepiploic artery and the planned anastomotic site.

**Results:**

Of 100 included patients, AL occurred in nine patients. Although there was no significant difference in ICG enhancement time between the AL and non-AL groups, fluorescence intensity at both the end of the right gastroepiploic artery (75 *versus* 101%; *P* = 0.004) and the anastomotic line (67 *versus* 90%; *P* = 0.009) was significantly lower in patients who developed AL. Multivariable analysis identified tumour location in the upper oesophagus and fluorescence intensity ≤ 90% at the anastomotic site as independent predictors of AL, with odds ratios of 6.99 (*P* = 0.023; 95% confidence interval (c.i.) 1.31 to 37.30) and 12.50 (*P* = 0.004; 95% c.i. 2.15 to 72.9), respectively.

**Conclusion:**

Quantitative ICG fluorescence intensity assessment facilitates objective intraoperative evaluation of gastric conduit perfusion and may support optimal anastomotic site selection, potentially reducing AL risk.

## Teaser

This prospective study investigated the relationship between intraoperative indocyanine green fluorescence intensity (FI) and anastomotic leakage (AL) in 100 patients undergoing oesophagectomy with gastric conduit reconstruction. Quantitative FI measured at the end of the right gastroepiploic artery and the anastomotic line was significantly lower in patients who developed AL, with FI ≤ 90% identified as an independent predictor. The findings suggest that real-time FI assessment can help optimize anastomotic site selection and reduce AL risk.

## Introduction

Oesophagectomy with gastric conduit reconstruction remains the standard curative treatment for oesophageal cancer. Despite significant advances in surgical techniques and perioperative care, oesophagectomy continues to be a highly invasive procedure associated with a high incidence of postoperative complications^1,2^.

Among the postoperative complications, anastomotic leakage (AL) is particularly concerning because it can lead to severe infections, prolonged hospitalization, and increased postoperative mortality^3,4^. Furthermore, accumulating evidence indicates that AL adversely affects both recurrence-free and overall survival following oesophagectomy^5–8^. Therefore, preventing AL is a critical objective in improving surgical outcomes. One of the major risk factors for AL is insufficient perfusion of the gastric conduit^9^. To assess blood flow in the conduit, techniques such as laser Doppler flowmetry^10–13^ and indocyanine green (ICG) fluorescence angiography^14^ have been used. Several studies have demonstrated that intraoperative ICG assessment significantly reduces the incidence of AL^15–18^. Moreover, quantitative parameters, such as the time to fluorescence and the rate of staining, have been proposed as objective measures for evaluating gastric conduit perfusion with ICG^16,19–21^.

However, no study to date has established objective indicators of ICG fluorescence for intraoperative determination of the anastomotic site in real time. In addition, the relationship between fluorescence intensity (FI), as a quantitative perfusion marker, and the occurrence of AL in gastrointestinal surgery, including oesophagectomy, has not been fully elucidated. Therefore, this study investigated the association between ICG FI and the incidence of AL in patients undergoing subtotal oesophagectomy with gastric conduit reconstruction, using a new real-time quantitative blood flow assessment method.

## Methods

### Patients

Patients with oesophageal cancer scheduled to undergo oesophagectomy at the Department of Esophageal Surgery, National Cancer Center Hospital East, Tokyo, Japan, were screened for study inclusion. The exclusion criteria were the absence of ICG assessment, reconstruction using the colon, two-stage reconstruction procedures, R1 or R2 resection status, and screen contamination.

Clinicopathological data were extracted from the patients’ medical records. Clinical staging was performed using endoscopy and computed tomography, and the clinical or pathological stage was determined according to the 8th edition of the Union for International Cancer Control (UICC) classification system^22^. The study was approved by the Ethics Committee of the National Cancer Center Hospital East (Approval no. 2018-322), and written informed consent was obtained from all participants for ICG administration and intraoperative fluorescence imaging.

### Quantification using SPY-PHI QP software

**Fig. 1:**
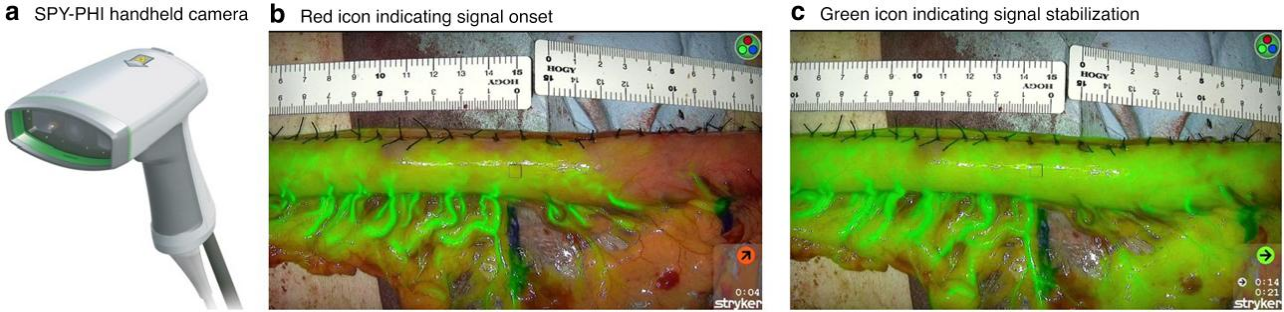
Intraoperative fluorescence imaging using the SPY-PHI system with SPY-QP software **a** The SPY-PHI handheld camera (Stryker AB, Malmö, Sweden) from https://www.stryker.com/jp/ja/portfolios/medical-surgical-equipment/surgical-visualization.html. **b** When fluorescence is first detected after indocyanine green injection, a red icon appears on the SPY-QP monitor to indicate the onset of the signal. **c** A green icon appears once the fluorescence signal has stabilized, enabling quantitative measurement of fluorescence intensity.

**Fig. 2:**
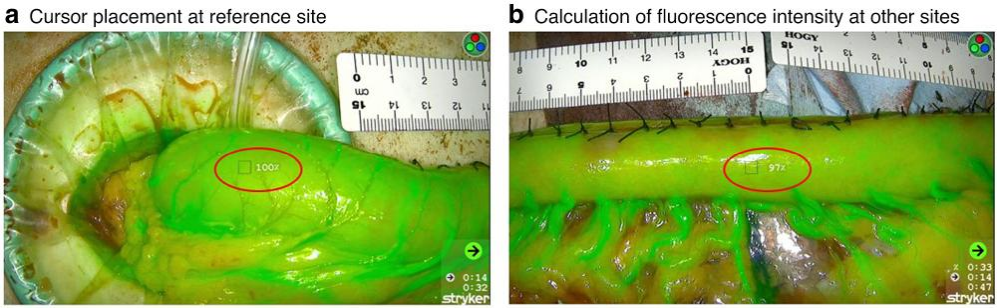
Quantitative assessment of gastric conduit perfusion with SPY-QP software **a** The cursor was placed at the point where the first branch of the right gastroepiploic artery entered the gastric conduit; this was designated as the reference site (100% perfusion). **b** Fluorescence intensity at other sites, including the anastomotic line, was calculated in real time and expressed as a relative percentage compared with the reference point.

Intraoperative fluorescence imaging was conducted using the SPY-PHI system equipped with SPY-QP software (Stryker AB, Malmö, Sweden), which enables visualization of fluorescent agents from a distance of up to 40 cm from the surgical field. The software facilitates quantitative evaluation of ICG FI by analysing captured data. When fluorescence is first detected, a red icon appears on the SPY-QP monitor to indicate its onset; a green icon then appears once the fluorescence signal has stabilized (*Fig. 1*). A reference point corresponding to 100% perfusion is designated using the cursor, after which the FI of other regions is measured in real time as a relative percentage once the fluorescence signal has stabilized (*Fig. 2*).

### Surgical procedure

Oesophagectomy with mediastinal lymphadenectomy was performed via a thoracoscopic or mediastinoscopic approach, including robotic-assisted techniques^23,24^. For the abdominal phase, laparoscopy (also with robotic assistance) was used, except in patients with bulky lymph node metastases or a history of abdominal surgery. Vascular dissection involved ligation of the left gastroepiploic, left gastric, and short gastric arteries, while preserving branches of the right gastroepiploic artery (RGEA) and right gastric artery to maintain adequate perfusion to the gastric conduit. The gastric conduit was designed as a narrow tube, beginning with stapling using a curved radial stapler (Endo GIA™ Radial Reload with Tri-Staple™; Medtronic, Minneapolis, MN, USA), followed by sequential application of linear staplers (Endo GIA™ with Tri-Staple™; Medtronic). The conduit was then elevated to the cervical region via either the retrosternal or posterior mediastinal route. The retrosternal route was used routinely; however, the posterior mediastinal route was selected when the retrosternal path was contraindicated because of previous surgical or interventional procedures involving the heart or liver. Cervical oesophagogastric anastomosis was primarily performed using the totally mechanical Collard technique^25^, using a 45-mm linear stapler for the posterior wall and a combination of 45- and 60-mm linear staplers for the anterior wall. When elevation of the gastric conduit was insufficient to facilitate this approach, alternative anastomotic methods, including circular stapling or hand-sewn techniques, were used. The anastomotic site and methods were not changed based solely on FI values. In all patients, a nasogastric tube was placed during surgery for anastomotic decompression.

### Intraoperative protocol of ICG fluorescence imaging

**Fig. 3:**
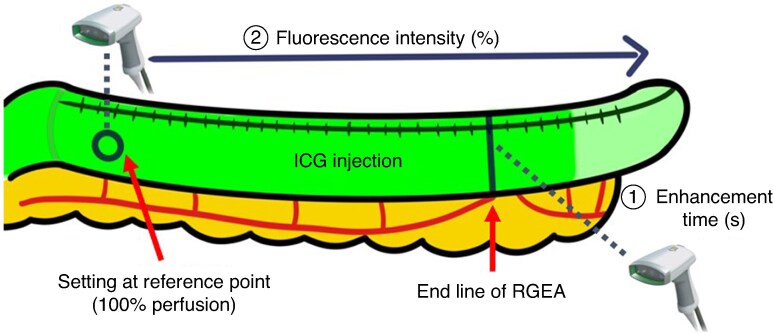
Intraoperative protocol for quantitative fluorescence assessment of the gastric conduit Following intravenous ICG injection, the enhancement time (①) was measured as the interval until fluorescence appeared at the distal end of the RGEA. After stabilization of the signal, fluorescence intensity was measured from the pylorus to the tip of the gastric conduit (②). The anterior wall at the entry point of the first RGEA branch was designated as the reference site, representing 100% perfusion. ICG, indocyanine green; RGEA, right gastroepiploic artery; s, seconds.

Evaluation of gastric conduit perfusion using ICG was performed before conduit elevation. A bolus of 6.25 mg ICG dye (Diagnogreen; Daiichi-Sankyo Pharmaceutical, Tokyo, Japan), followed by 20 ml saline, was administered via a peripheral vein in the upper limb. Fluorescence signals were captured using the SPY-PHI system equipped with SPY-QP software (Stryker AB), and real-time imaging data were recorded. The hand-held camera was positioned 20 cm above the gastric conduit. The interval between ICG injection and fluorescence appearance at the distal end of the RGEA was measured in seconds (s). After stabilization of the fluorescence signal was confirmed, the FI from the pylorus to the tip of the gastric conduit was assessed and recorded (*Fig. 3*; *Video S1*). The central point of the anterior wall, where the first branch of the RGEA entered the gastric conduit, was designated as the reference point. This point represented 100% perfusion because the RGEA was considered the main source of blood supply to the conduit (*Fig. 2*)^26^. Once the FI across the conduit had been confirmed, the gastric conduit was elevated to the cervical field. The anastomotic site was determined according to the conduit reach and the planned anastomotic technique, and the FI at the anastomotic line was assessed from the video (*Video S1*).

### Postoperative management

Following surgery, all patients were extubated in the operating room and subsequently transferred to the intensive care unit. On postoperative day (POD) 1, enteral nutrition was initiated via enterostomy or gastroenterostomy. On POD 2, patients were discharged from the intensive care unit and transferred to the general ward. On POD 6, swallowing angiography was performed under fluoroscopic guidance to evaluate anastomotic integrity and lumen patency. In the absence of AL, the nasogastric tube was removed and oral intake was started. Oral caloric intake was gradually increased, while tube feeding was correspondingly tapered based on the patient's oral intake capacity. Patients were discharged once their overall condition was deemed clinically stable.

### Definition of postoperative complications

Postoperative complications were classified according to the Clavien–Dindo grading system^27^, with complications of grade I or higher defined as postoperative events. AL was defined by the presence of clinical signs of salivary fistula and confirmed via either a water-soluble contrast swallow study under fluoroscopy or endoscopic visualization of dehiscence or fistula at the anastomotic site^28^.

### Statistical analysis

All statistical analyses were conducted using EZR (Saitama Medical Center, Jichi Medical University, Saitama, Japan)^29^. Categorical variables were compared using Fisher's exact test, whereas continuous variables were compared using Student's *t*-test. The Mann–Whitney *U* test was used to assess associations between continuous variables derived from ICG fluorescence imaging and the occurrence of AL. Receiver operating characteristic (ROC) curve analysis was performed to determine the optimal ICG FI cut-off value. Risk factors for AL were identified through univariable and multivariable logistic regression analyses, with significant factors in univariable analysis included in the multivariable model. Two-tailed *P* < 0.050 was considered statistically significant.

## Results

### Clinical characteristics

**Fig. 4:**
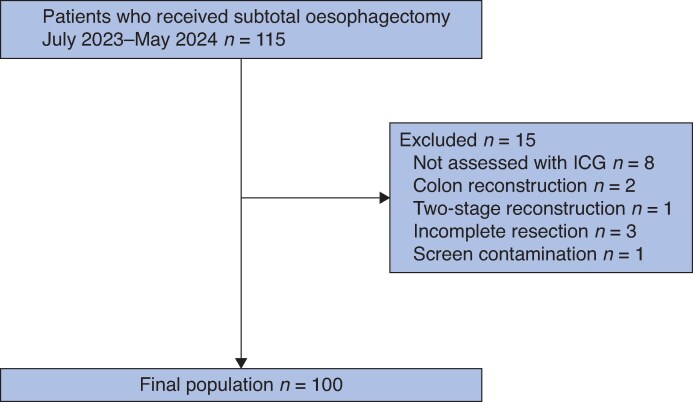
Patient selection diagram ICG, indocyanine green.

In all, 115 patients diagnosed with oesophageal cancer underwent subtotal oesophagectomy with lymphadenectomy at the Department of Esophageal Surgery, National Cancer Center Hospital East, Tokyo, Japan, between July 2023 and May 2024. The process of patient selection is shown in *Fig. 4*. After application of the exclusion criteria, 100 patients who underwent subtotal oesophagectomy followed by gastric conduit reconstruction with intraoperative ICG evaluation were prospectively included in this study. The clinical characteristics of the 100 patients are presented in *Table 1*. The median patient age was 69 (range 39–87) years, and 74% of patients were male. Tumours were located in the upper oesophagus (17%), middle oesophagus (44%), and lower oesophagus (39%). Clinical staging at diagnosis was stage I in 21% of patients, stage II in 12% of patients, stage III in 41% of patients, stage IVA in 6% of patients, and stage IVB in 20% of patients.

**Table 1:** Clinical characteristics of the 100 patients who underwent subtotal oesophagectomy with gastric conduit reconstruction

Age (years), median (range)	69 (39–87)
**Sex**	
Male	74 (74.0%)
Female	26 (26.0%)
BMI (kg/m^2^), median (range)	21.6 (15.8–38.0)
**Medical history**	
Respiratory distress	15 (15.0%)
Diabetes	14 (14.0%)
Hypertension	49 (49.0%)
Cerebrovascular disease	6 (6.0%)
Cardiovascular disease	13 (13.0%)
**Clinical tests, median (range)**	
Albumin (g/dl)	3.9 (1.5–5.4)
Haemoglobin (g/dl)	11.4 (8.0–15.2)
HbA1c (%)	5.7 (4.4–9.7)
PNI, median (range)	46.3 (21.5–59.7)
**Tumour location**	
Upper	17 (17.0%)
Middle	44 (44.0%)
Lower	39 (39.0%)
**Histology**	
Squamous cell carcinoma	85 (85.0%)
Adenocarcinoma	14 (14.0%)
Neuroendocrine carcinoma	1 (1.0%)
**cStage**	
cStage I	21 (21.0%)
cStage II	12 (12.0%)
cStage III	41 (41.0%)
cStage IVA	6 (6.0%)
cStage IVB	20 (20.0%)
**Preoperative treatment**	
Chemotherapy	68 (68.0%)
Chemoradiotherapy	9 (9.0%)
None	23 (23.0%)

Values are n (%) unless otherwise stated. BMI, body mass index; PNI, prognostic nutritional index.

### Operative outcome

Operative details are summarized in *Table 2*. All patients underwent minimally invasive oesophagectomy via either a thoracoscopic (82%) or mediastinoscopic (18%) approach, both of which included robotic-assisted procedures. Robotic assistance was used in 75.6 and 83.3% of thoracoscopic and mediastinoscopic procedures, respectively. The retrosternal route was used for reconstruction in 98 patients. Cervical anastomosis was achieved using the totally mechanical Collard technique in 88 patients and a circular stapler in 10 patients. Hand-sewn anastomosis was performed in two patients as a result of either poor elevation of the gastric conduit or insufficient residual cervical oesophagus length, which precluded the use of mechanical anastomosis.

**Table 2:** Operative outcomes of the 100 patients who underwent subtotal oesophagectomy with gastric conduit reconstruction

Operative time (min), median (range)	451 (253–656)
Blood loss (ml), median (range)	112 (22–742)
**Surgical procedure**	
Thoracoscopic	82 (82.0%)
Mediastinoscopic	18 (18.0%)
**Lymphadenectomy**	
Two-field	32 (32.0%)
Three-field	68 (68.0%)
**Route of reconstruction**	
Retrosternal	98 (98.0%)
Posterior mediastinal	2 (2.0%)
**Anastomotic procedure**	
Totally mechanical Collard	88 (88.0%)
Circular stapler	10 (10.0%)
Hand-sewn	2 (2.0%)

Values are *n* (%) unless otherwise stated.

### Postoperative outcomes

Postoperative outcomes are summarized in *Table 3*. AL occurred in nine patients, and gastric conduit necrosis was observed in one patient with severe diabetes (FI at the end of the RGEA was measured as 12% in this patient). Surgical site infection was documented in nine patients. Eleven patients developed postoperative pneumonia. The median length of hospitalization after surgery was 17 days. There were no in-hospital deaths.

**Table 3:** Postoperative outcomes in patients who underwent subtotal oesophagectomy with gastric conduit reconstruction

Anastomotic leakage	9 (9.0%)
Necrosis of gastric conduit	1 (1.0%)
Pneumonia	11 (11.0%)
Recurrent nerve paralysis	16 (16.0%)
Surgical site infection	9 (9.0%)
Postoperative hospital stay (days), median (range)	17 (10–141)
In-hospital mortality	0 (0%)

Values are *n* (%) unless otherwise stated.

### ICG fluorescence imaging of the gastric conduit

**Fig. 5:**
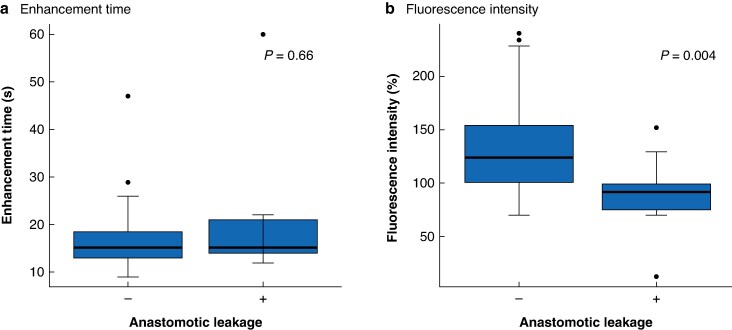
Quantitative fluorescence assessment at the distal end of the right gastroepiploic artery **a** Indocyanine green enhancement time did not differ significantly between patients with (+) and without (–) anastomotic leakage (*P* = 0.66). **b** Fluorescence intensity was significantly lower in patients with than without anastomotic leakage (*P* = 0.004). Boxes show interquartile range, with median value indicated by the horizontal line; whiskers show the range. Black dots represent outliers beyond 1.5 x interquartile range from the box limits. s, seconds.

**Fig. 6:**
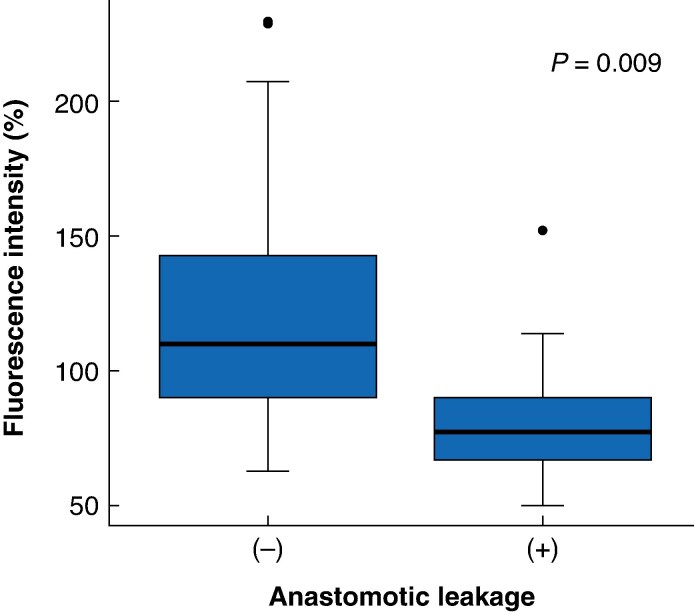
Fluorescence intensity at the anastomotic line Indocyanine green fluorescence intensity was significantly lower in patients with than without anastomotic leakage (*P* = 0.009). Boxes show interquartile range, with median value indicated by the horizontal line; whiskers show the range. Black dots represent outliers beyond 1.5 x interquartile range from the box limits.

*Figure 5* shows two box plots comparing the ICG enhancement time and FI between patients with and without AL at the end of the RGEA. There was no significant difference in enhancement time between patients without and with AL (13 *versus* 14 s, respectively; *P* = 0.660; *Fig. 5a*). However, FI was significantly higher in patients without than with (101 *versus* 75%, respectively; *P* = 0.004; *Fig. 5b*). Similarly, at the anastomotic line, FI was significantly greater in the group without than with AL (90 *versus* 67%, respectively; *P* = 0.009; *Fig. 6*).

### Risk assessment for AL

**Fig. 7:**
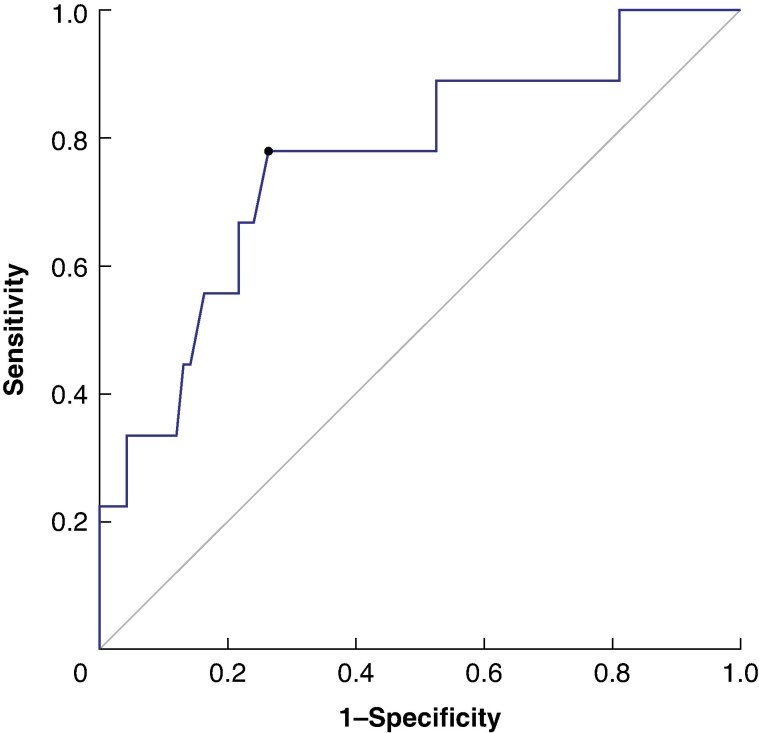
Receiver operating characteristic curve for predicting anastomotic leakage Indocyanine green fluorescence intensity at the anastomotic line was measured using SPY-QP software, with 100% perfusion defined at the anterior wall where the first branch of the right gastroepiploic artery entered the gastric conduit. A fluorescence intensity cut-off value of 90% at the anastomotic line yielded an area under the curve of 0.76 for the prediction of anastomotic leakage.

Risk factors associated with AL were investigated using univariable and multivariable logistic regression analyses. ROC curve analysis was conducted to assess the predictive value of ICG FI at the anastomotic line for AL (*Fig. 7*). An FI cut-off value of 90% yielded an area under the curve of 0.76 (95% confidence interval 0.58 to 0.95). In univariable analysis, tumour location (upper *versus* middle/lower oesophagus: odds ratio (OR) 4.80; *P* = 0.033) and ICG FI at the anastomotic line (≤ 90 *versus* > 90%: OR 9.770; *P* = 0.006) were significantly associated with AL. Multivariable analysis confirmed tumour location in the upper oesophagus and ICG FI ≤ 90% at the anastomotic site as independent risk factors for the development of AL, with ORs of 6.990 (*P* = 0.023) and 12.50 (*P* = 0.004), respectively (*Table 4*).

**Table 4:** Univariable and multivariable analyses of factors associated with anastomotic leakage in patients who underwent subtotal oesophagectomy with gastric conduit reconstruction

	Univariable analysis		Multivariable analysis	
	OR*	*P*	OR*	*P*
Age ≥ 69 years (*versus* <69 years)	1.22 (0.31, 4.85)	0.78		
Sex: male (*versus* female)	1.25 (0.24, 6.45)	0.79		
BMI ≥ 21.6 kg/m^2^ (*versus* <21.6 kg/m^2^)	0.78 (0.20, 3.10)	0.73		
Albumin ≤ 3.9 g/dl (*versus* >3.9 g/dl)	0.72 (0.18, 2.84)	0.64		
Haemoglobin ≤ 11.4 g/dl (*versus* >11.4 g/dl)	0.45 (0.11, 1.90)	0.28		
HbA1c ≥ 5.7% (*versus* <5.7%)	0.72 (0.18, 2.84)	0.64		
PNI ≤ 46.3 (*versus* > 46.3)	1.28 (0.32, 5.07)	0.73		
Tumour location: upper (*versus* middle/lower)	4.80 (1.14, 20.30)	0.033	6.99 (1.31, 37.30)	0.023
cT3–4 (*versus* cT1–2)	0.84 (0.20, 3.62)	0.82		
cN1–3 (*versus* cN0)	0.8 (0.19, 3.44)	0.76		
CRT present (*versus* absent)	1.3 (0.14, 11.7)	0.82		
Operative time ≥ 451 min (*versus* <451 min)	1.02 (0.26, 4.05)	0.98		
Blood loss ≥ 112 ml (*versus* <112 ml)	2.04 (0.48, 8.68)	0.33		
Surgical procedure: mediastinoscopic (*versus* thoracoscopic)	1.34 (0.25, 7.06)	0.73		
Route of reconstruction: posterior mediastinal (*versus* retrosternal)	0 (0, Infinity)	0.99		
Anastomotic procedure: circular stapler/hand-sewn (*versus* totally mechanical Collard)	4.56 (0.97, 21.40)	0.055		
ICG fluorescence intensity in the anastomotic line ≤ 90% (*versus* > 90%)	9.77 (1.90, 50.30)	0.006	12.50 (2.15, 72.9)	0.004

*Values in parentheses are 95% confidence intervals. OR, odds ratio; BMI, body mass index; PNI, prognostic nutritional index; CRT, chemoradiotherapy; ICG, indocyanine green.

## Discussion

This study examined the association between intraoperative ICG FI and the risk of AL in patients undergoing subtotal oesophagectomy with gastric conduit reconstruction, with the aim of evaluating the clinical utility of a quantitative blood flow assessment technique for the conduit. Assessment of gastric conduit perfusion with ICG is associated with a reduced incidence of AL^15–,18^. However, no study has yet established objective ICG fluorescence criteria derived from statistical analysis for real-time determination of the anastomotic site during surgery. In the present study, lower ICG FI at both the terminal branch of the RGEA and the anastomotic site of the gastric conduit was significantly correlated with AL. Specifically, FI ≤ 90% at the anastomotic site was identified as an independent risk factor for AL. These findings suggest that real-time quantitative blood flow assessment using ICG may be valuable for predicting and potentially preventing AL.

Kitagawa *et al*.^20^ previously reported an association between delayed ICG arrival at the tip of the gastric conduit and the development of AL. However, the absence of fluorescence at the distal conduit in some patients and variability in conduit length limited the objectivity of their evaluation, which was inherently subjective. Campbell *et al*.^15^ demonstrated that the use of the SPY Elite System to evaluate ICG FI reduced the incidence of AL, although their study did not specifically quantify FI at the gastric conduit. As such, previous methods using ICG have lacked the precision needed for accurate perfusion assessment. In line with the approach described in the present study, Nerup *et al*.^30^ demonstrated the feasibility and practicality of real-time intraoperative quantitative ICG perfusion assessment during gastroesophageal junction cancer resection, highlighting the methodological relevance of incorporating objective quantitative perfusion metrics into surgical practice. The present study introduces an objective evaluation of gastric conduit perfusion by measuring both enhancement time and FI at the terminal point of the RGEA, the primary feeding artery^26^.

Importantly, visual evaluation of ICG fluorescence alone is inherently subjective and dependent on the surgeon's interpretation of colour intensity. This limitation has been highlighted by Nerup *et al*.,^31^ who emphasized the need to quantify ICG fluorescence rather than rely solely on visual inspection. Consistent with this, the findings of the present study indicate that FI, rather than enhancement time or visual appearance, is a more reliable and objective predictor of AL. Quantitative assessment provides a reproducible indicator that minimizes interobserver variability and enables real-time intraoperative decision-making regarding anastomotic site selection.

Impaired perfusion in these distal branches may directly reflect inadequate blood supply to the anastomotic site, increasing the likelihood of tissue ischaemia and subsequent AL. Interestingly, no significant differences in enhancement time were observed between patients with and without AL. This suggests that the timing of fluorescence onset alone is not a reliable predictor of anastomotic perfusion, particularly given that interpretation of serosal membrane staining remains subjective. In contrast, FI values at both the RGEA termination and the anastomotic line were significantly associated with AL. These data underscore the critical role of insufficient perfusion, quantified objectively via intraoperative ICG FI, in the pathogenesis of AL. The findings of the present study support the clinical utility of ICG FI as a quantitative marker for real-time assessment of perfusion during oesophagectomy. Specifically, the study identified a cut-off value of 90% FI at the anastomotic site, which may serve as a threshold for identifying areas of compromised blood flow. This allows surgeons to reconsider or reinforce the anastomosis during the surgery to reduce the risk of AL. In some patients, FI exceeded 100% in certain areas of the gastric conduit. This was considered to reflect differences in wall thickness: because the pylorus, which was used as the reference point, was relatively thick, FI values exceeded 100% in thinner regions of the conduit.

Previous reports have identified upper thoracic tumours as significant risk factors for AL in patients undergoing oesophagectomy with cervical anastomosis^32^. Consistent with previous findings, the present study demonstrated that tumour location in the upper thoracic region was an independent risk factor for AL. It is hypothesized that the short length of the residual cervical oesophagus contributes to increased tension at the anastomotic site, thereby elevating the risk of leakage. In patients requiring a high-level anastomosis, it is essential to adopt techniques that minimize anastomotic tension. Surgeons should aim to preserve as much length of the residual oesophagus as oncologically permissible, ensuring adequate negative margins. Furthermore, the anastomotic height of the gastric conduit can be increased by using intraoperative ICG FI to guide conduit elevation and by optimizing mobilization techniques such as Kocher's manoeuvre. In contrast, the circular stapler and hand-sewn anastomotic techniques showed a trend towards statistical significance in relation to AL. This was likely because these anastomotic procedures were performed in patients with tumours located in the upper thoracic region.

The present study has certain limitations. First, it was conducted at a single centre and had a relatively small sample size. Therefore, multicentre studies with larger cohorts are warranted to validate the clinical utility of ICG FI and to refine the threshold values predictive of AL. Second, the quantitative perfusion of the residual cervical oesophagus was not evaluated. It is possible that inadequate blood supply to both the gastric conduit and the residual oesophagus contributes to the development of AL. Future investigations will include evaluation of ICG FI in the cervical oesophagus to provide a more comprehensive assessment. Third, while the findings establish an association between reduced FI and AL, additional studies are needed to determine whether intraoperative adjustments based on FI can proactively decrease the incidence of AL. Furthermore, it is hoped that, in the future, artificial intelligence will be able to determine the optimal anastomotic range based on real-time FI values of the gastric conduit when selecting the anastomosis site.

In conclusion, the findings of this study suggest that real-time quantitative assessment of ICG FI may facilitate prediction and reduction of AL following oesophagectomy. An FI threshold of 90% at the anastomotic line appears to be a clinically relevant intraoperative indicator for guiding optimal site selection. This new approach to quantitatively assessing gastric conduit perfusion with ICG FI represents a potentially effective strategy for mitigating the risk of AL during oesophagectomy.

## Supplementary Material

zraf135_Supplementary_Data

## Data Availability

The data sets used and/or analysed during the present study are available from the corresponding author upon reasonable request.
